# Subanesthetic ketamine rapidly alters medial prefrontal miRNAs involved in ubiquitin-mediated proteolysis

**DOI:** 10.1371/journal.pone.0256390

**Published:** 2021-08-26

**Authors:** Yunjung Choi, Baeksun Kim, Suji Ham, Sooyoung Chung, Sungho Maeng, Hye-Sun Kim, Heh-In Im

**Affiliations:** 1 Convergence Research Center for Diagnosis, Treatment and Care System of Dementia, Korea Institute of Science and Technology (KIST), Seoul, South Korea; 2 Department of Pharmacology, College of Medicine, Seoul National University, Seoul, South Korea; 3 Division of Bio-Medical Science & Technology, KIST School, Korea University of Science and Technology, Seoul, South Korea; 4 Center for Neuroscience, Brain Science Institute, Korea Institute of Science and Technology, Seoul, South Korea; 5 College of East-West Medical Science, Kyung Hee University, Yongin, South Korea; 6 Department of Pharmacology, Seoul National University Bundang Hospital, Seongnam, Bundang-Gu, South Korea; Chiba Daigaku, JAPAN

## Abstract

Ketamine is a dissociative anesthetic and a non-competitive NMDAR antagonist. At subanesthetic dose, ketamine can relieve pain and work as a fast-acting antidepressant, but the underlying molecular mechanism remains elusive. This study aimed to investigate the mode of action underlying the effects of acute subanesthetic ketamine treatment by bioinformatics analyses of miRNAs in the medial prefrontal cortex of male C57BL/6J mice. Gene Ontology and KEGG pathway analyses of the genes putatively targeted by ketamine-responsive prefrontal miRNAs revealed that acute subanesthetic ketamine modifies ubiquitin-mediated proteolysis. Validation analysis suggested that miR-148a-3p and miR-128-3p are the main players responsible for the subanesthetic ketamine-mediated alteration of ubiquitin-mediated proteolysis through varied regulation of ubiquitin ligases E2 and E3. Collectively, our data imply that the prefrontal miRNA-dependent modulation of ubiquitin-mediated proteolysis is at least partially involved in the mode of action by acute subanesthetic ketamine treatment.

## Introduction

Ketamine is a non-competitive N-methyl-D-aspartate receptor (NMDAR) antagonist that can induce dissociative anesthesia in humans [1]. Interestingly, when subanesthetic dose is administered, ketamine can exert pain relief and fast-acting antidepressant effects [1–3]. Studies have shown that depression patients and animal models exhibit immediate and significant improvement in depressive symptoms with only a single injection of subanesthetic ketamine [3, 4].

The leading hypothesis about the rapid subanesthetic effects of ketamine is based on NMDAR inhibition-mediated mechanisms [5]. Ketamine blocks NMDAR to inhibit the flow of calcium ions, which eventually decreases the firing of the neurons expressing NMDAR. However, controversy exists among studies on the type of neurons that ketamine exerts its effects. Substantial evidence have suggested that the subanesthetic dose of ketamine preferentially blocks NMDAR on GABAergic interneurons, consequently lifting the inhibition on excitatory neurons [6–8]. Accordingly, ketamine increases the glutamate level and overall activity in the prefrontal cortex [9, 10], which is thought to underlie the antidepressant effects of ketamine. However, ketamine has also been demonstrated to be capable of blocking NMDAR in the presynaptic glutamatergic neuron and consequently inhibits burst activity of post-synaptic neuron in depressed animals [11], which is consistent with the finding that ketamine normalizes hyperactivity in the prefrontal cortex of depressed patients [3]. Thus, the mechanisms of ketamine mediated by NMDAR antagonism are mutually exclusive, demanding a new avenue to explore.

MicroRNAs (miRNAs) are short, non-coding RNAs that regulate gene expression by suppressing translation of messenger RNAs (mRNAs) or causing mRNA degradation [12]. Studies have reported that miRNAs are dynamically altered in the brain after ketamine treatment [13–15]. In addition, expression of miRNAs is capable of changing in minutes and rapidly regulating their targets [16, 17], which raises the possibility that miRNAs mediate the fast-acting effects of ketamine. However, the role of miRNAs in the rapid effects of acute subanesthetic ketamine has yet to be investigated.

Medial prefrontal cortex (mPFC) is a core brain region involved in emotion regulation [18] and pain sensation [19]. Interestingly, a study reported that systemic ketamine is associated with increased dendritic spine density in the mPFC of naïve rodents [20]. Also, repeated stress induces loss of dendritic spines in the rat mPFC [21]. Therefore, our study focused on the mPFC as the brain region responsible for the effects induced by acute subanesthetic ketamine administration.

We placed emphasis on the prefrontal miRNAs that are altered by acute subanesthetic ketamine and on the bioinformatics analyses of the genes putatively targeted by ketamine-responsive miRNAs. We sought to discover a novel mode of action underlying acute subanesthetic ketamine by tracking the main molecular pathway targeted by the ketamine-responsive miRNAs in the mPFC.

## Materials and methods

### Mice

Two-month-old C57BL/6J male mice were purchased from Daehan Biolink (Chungbuk, Korea). The experiment was done after one-week adaptation. The mice were maintained in a temperature and humidity-controlled facility (22±2°C, 50±5%) with a 12-hour light/dark cycle. Food and water were provided *ad libitum*. Anesthetic dose of ketamine in mice is known to be 80~100mg/kg (i.p. or i.m.) [22–24], while subanesthetic dose of ketamine with effect is 10mg/kg (i.p.) [25–27]. Thus, for the ketamine-administered group, mice were intraperitoneally injected with subanesthetic dose of ketamine (10mg/10ml/kg). For the control group, mice were injected with the same volume of physiological saline. Mice were sacrificed by cervical dislocation 30 min after ketamine/saline treatment. All procedures regarding the use and the handling of the animals were conducted as approved by the Institutional Animal Care and Use Committee (IACUC) of Korea Institute of Science and Technology (#2015–009).

### mPFC RNA isolation and quality measurement

Mice were sacrificed 30 min after ketamine or vehicle injection. Frozen whole brains were cut into 100μm slices on the coronal plane with a cryostat (CM3050S, Leica Microsystems, Inc., Wetzlar, Germany) at -20°C. mPFC was micro-dissected and collected from the slices. Total RNA of each sample was isolated with Trizol reagent (Life Technologies, Inc., Carlsbad, CA, USA) following the manufacturer’s instruction. The RNA quantification was done using ND-1000 spectrophotometer (NanoDrop Technologies, Inc., Wilmington, DE, USA). The RNA quality was verified with 1% agarose denaturing gel and Agilent 2100 Bioanalyzer (Agilent Technologies, Palo Alto, CA, USA).

### miRNA microarray analysis

Total 1881 miRNA probes were synthesized and hybridized using miRNA Labeling Reagent and Hybridization kit (Agilent Technologies) according to the manufacturer’s instruction. The hybridized images were scanned using DNA microarray scanner and quantified with Feature Extraction Software (Agilent Technologies). Normalization of the data and selection of differentially expressed miRNAs were performed using GeneSpringGX 7.3 (Agilent Technologies). Two mice were used per group according to previous studies with microarray analysis [28, 29]. Raw data value over 5 was selected for stable analysis based on a previously published paper [30]. To further reduce false positives in the microarray data analysis, miRNAs with fold change lower than 10% were excluded. DESeq2 was employed to estimate the fold change of the count data, as this corrects for library size and improves the stability of variance [31].

### miRNA qPCR

miRNA qPCR was done for the validation of differentially expressed miRNAs in the miRNA microarray analysis. 10 mice were used per group. 50ng of total RNA was used from each sample for cDNA preparation through reverse transcription. cDNA was amplified using TaqMan Universal Master Mix II (Life Technologies, Inc., Carlsbad, CA, USA) following the manufacturer’s protocol. Thereafter, expression of the miRNAs was quantified using quantitative real-time PCR (qPCR) with TaqMan MicroRNA Assays (Life Technologies, Inc., Carlsbad, CA, USA). qPCR reactions were run on CFX connect (Bio-Rad, Hercules, CA, USA). All reactions were performed in triplicates. The relative abundance of miRNAs was calculated using the 2^-ddCt^ method. snoRNA202 was used as a normalization control.

### Statistics

For qPCR data, statistical analysis was done with multiple t-test (GraphPad Prism, San Diego, CA). For miRNA microarray data, bootstrap analysis (random sampling with replacement) was performed to identify differentially expressed miRNAs [32]. Bootstrap p-value was obtained for each miRNA from 100,000 repetitions of bootstrap dataset analysis. P-value under 0.05 was considered statistically significant.

### Integrated analysis of the miRNA targets and bioinformatic analysis

TargetScan v7.2 was used to predict the targets of miRNAs [33], in which genes with cumulative weighted context++ score under -0.3 were selected as putative targets. miRNAs that exhibited different expression pattern from other miRNAs within the same miRNA cluster were excluded from analysis [34]. For gene ontology (GO) analysis, Functional Annotation Clustering tool in the Database for Annotation, Visualization and Integrated Discovery (DAVID) v6.8 was used [35]. Annotation clusters with Enrichment Score over 2 were considered as significantly altered. The annotation clusters consisted of GO terms within 3 aspects: biological process, cellular component and molecular function. To identify the functional pathways associated with the genes putatively targeted by miRNAs, we employed the Kyoto Encyclopedia of Genes and Genomes (KEGG, http://www.genome.ad.jp/kegg) enrichment analysis [36]. KEGG pathways with -log_10_(p-value) value over 2 were considered as significantly altered.

## Results

### Five miRNAs are rapidly altered in the mPFC of mice by subanesthetic ketamine administration

**Fig 1:**
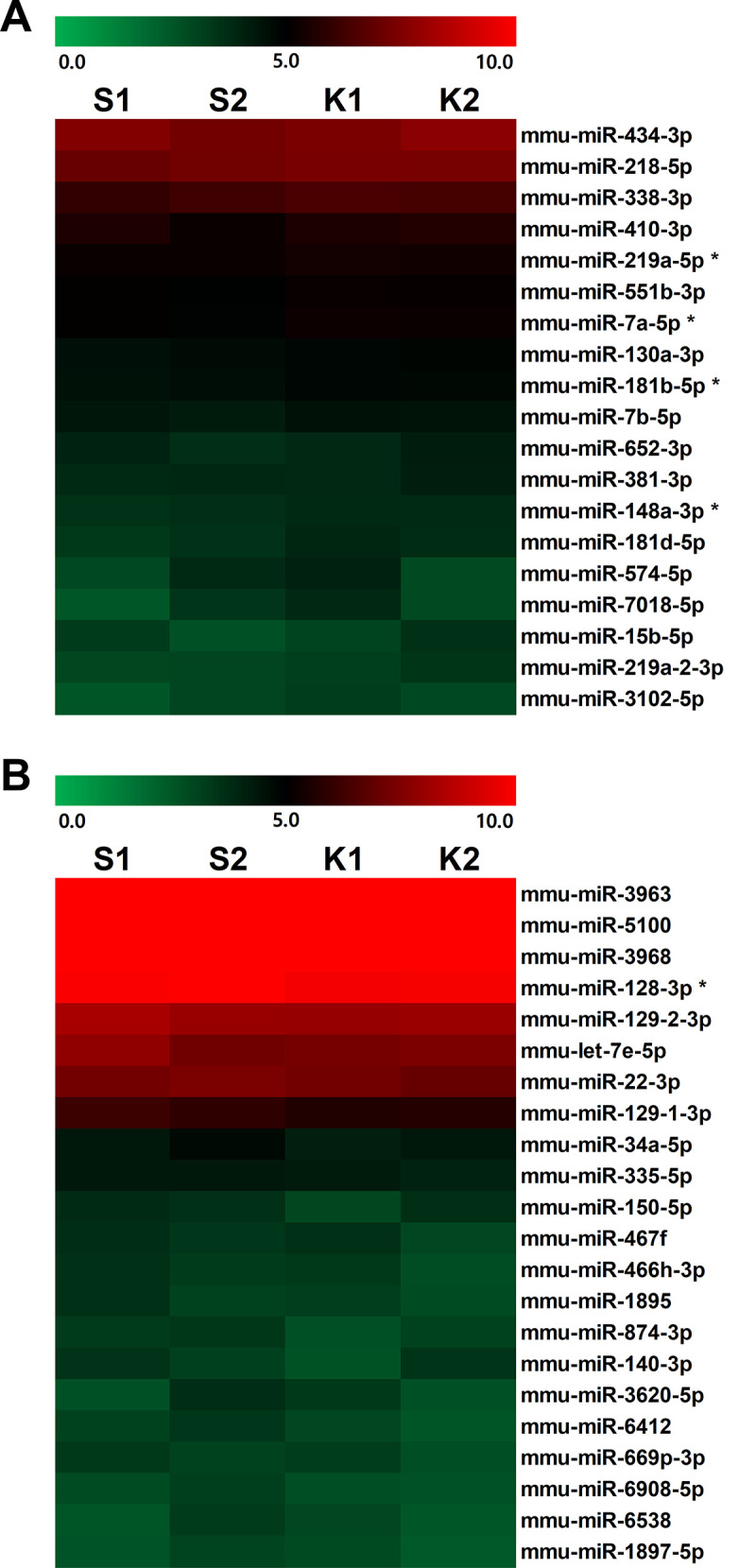
Prefrontal miRNA expression profiles in acute subanesthetic ketamine-administered mice. The heatmap of miRNA expression levels with raw data values over 5 with fold change over 10% in the medial prefrontal cortex. Prefrontal miRNAs with (A) upregulated and (B) downregulated expression patterns in ketamine-treated mice. The order of miRNAs is sorted by expression level. Stars mark five miRNAs that are significantly altered.

To confirm the role of miRNAs in the fast-acting effect of ketamine, mice were sacrificed 30 min after ketamine treatment. The mPFC of ketamine-treated group exhibited differential expression profiles of miRNAs by acute subanesthetic ketamine. Among 1881 miRNAs analyzed, a total of 41 miRNAs were found to have stable value of raw data (raw count >5) with fold change over 10%. A total 19 miRNAs were upregulated (Fig 1A) and 22 miRNAs were downregulated (Fig 1B) in the ketamine-treated mice. Screening for miRNAs with p-value under 0.05 (bootstrap), we identified five miRNAs that were differentially expressed in response to acute subsanesthetic ketamine treatment. The final five ketamine-responsive miRNAs are listed in Table 1.

**Table 1:** Differentially expressed prefrontal miRNAs by subanesthetic ketamine treatment.

miRNA	Saline	Ketamine	%Change	P-Value
mmu-miR-128-3p	994.945	894.469	-10.099	0.04679
mmu-miR-219a-5p	36.823	40.902	11.079	0.04682
mmu-miR-7a-5p	32.026	37.603	17.415	0.04690
mmu-miR-181b-5p	24.039	28.126	17.005	0.04702
mmu-miR-148a-3p	12.438	14.240	14.486	0.04723

Values in Saline and Ketamine represent raw count.

### Genes targeted by the ketamine-responsive miRNAs are involved in ubiquitin-mediated proteolysis

**Fig 2:**
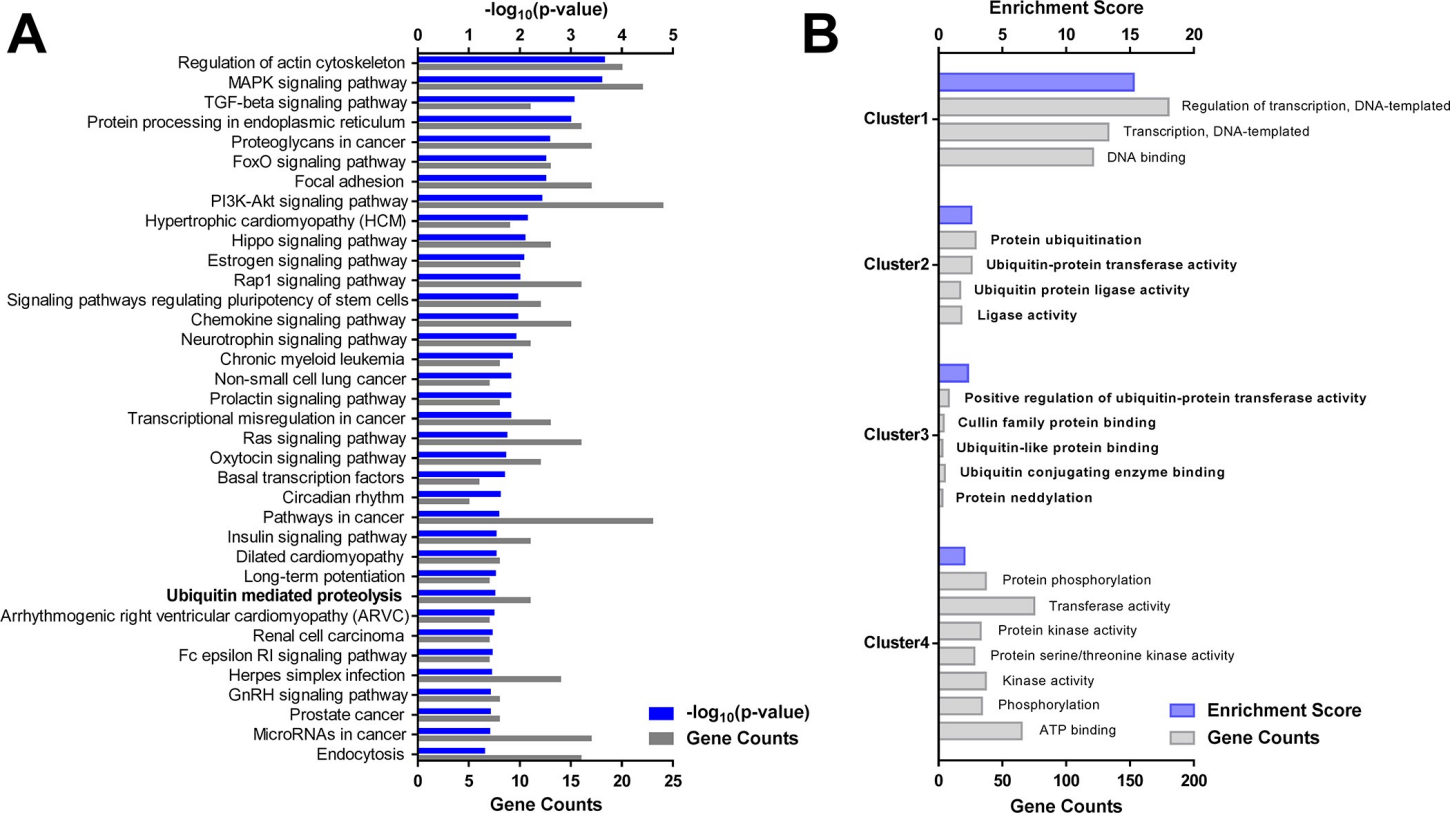
Functional annotation clustering of GO terms and KEGG pathways from the genes targeted by the ketamine-responsive prefrontal miRNAs. (A) KEGG pathways with p-values under 0.05 are displayed. The vertical axis represents the pathway categories, the upper horizontal axis represents -log_10_(p-value) value of each pathway, and the lower horizontal axis represents the number of genes involved in each pathway. (B) Functional annotation clusters with Enrichment Scores over 2 are displayed. The vertical axis represents the GO terms grouped into four clusters, the upper horizontal axis represents enrichment score values of each GO term, and the lower horizontal axis represents the number of genes included in each GO term.

The five ketamine-responsive miRNAs were predicted to target a total of 781 genes based on the miRNA target database TargetScan 7.2 (S1 Table). KEGG pathways of the 781 genes targeted by ketamine-responsive prefrontal miRNAs were analyzed through DAVID functional annotation tool (Fig 2A). 36 molecular pathways were predicted to be altered by acute subanesthetic ketamine treatment. To cross-examine and identify significant pathways from the KEGG pathway analysis result, Gene Ontology (GO) of the 781 genes were analyzed through the DAVID functional annotation tool (Fig 2B). The genes targeted by miRNAs were grouped into four annotation clusters. The clusters were related to transcription, ubiquitin proteasome system, and phosphorylation. Notably, two out of four annotation clusters were concurrently associated with ubiquitin proteasome system (Fig 2B; Cluster 2 and 3), which overlapped with ubiquitin-mediated proteolysis screened in the KEGG pathway analysis. These results led us to the conclusion that ubiquitin proteasome system might be an important element for the mode of action of ketamine.

### miR-148a-3p and miR-128-3p are associated with E2- and E3-related genes in ubiquitin-mediated proteolysis pathway

**Fig 3:**
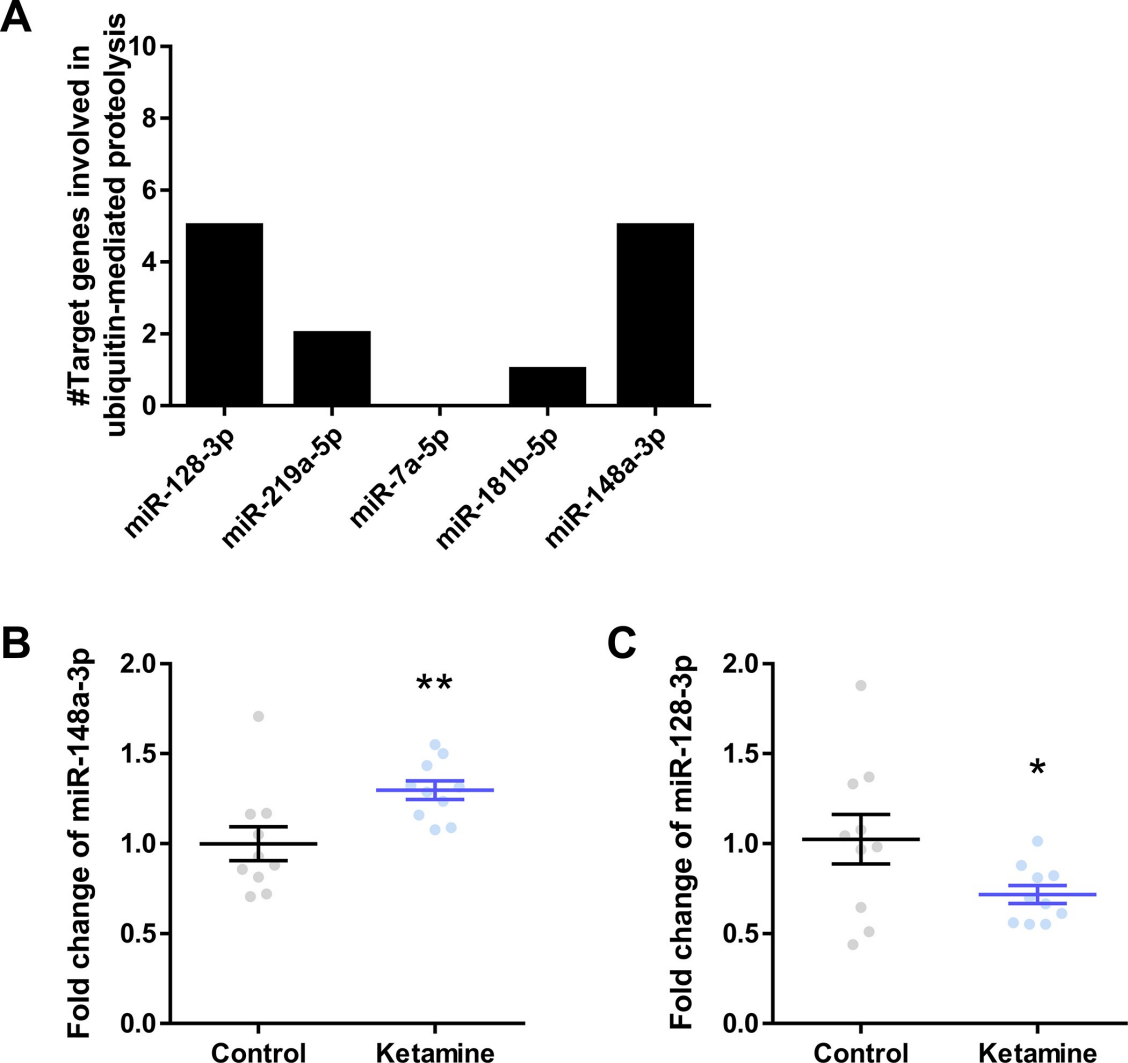
miR-148a-3p and miR-128-3p as the principal miRNA substrates for acute subanesthetic ketamine. (A) Number of genes involved in ubiquitin-mediated proteolysis targeted by the 5 miRNAs. The miRNA qPCR result of (B) miR-148a-3p and (C) miR-128-3p. *p-value<0.05, **p-value<0.01.

**Fig 4:**
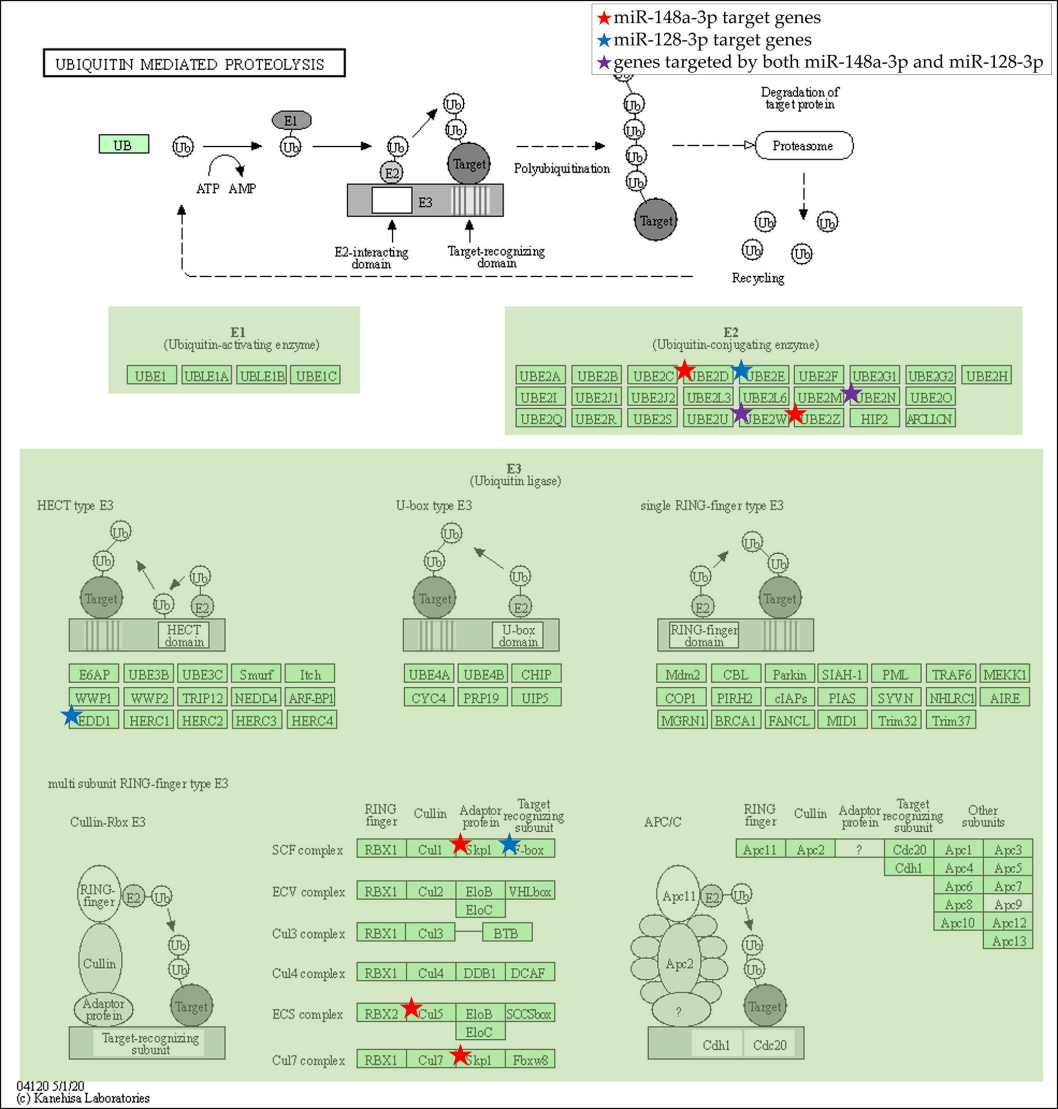
KEGG pathway map of ubiquitin-mediated proteolysis with the genes putatively targeted by miR-148a-3p and miR-128-3p. Genes marked with stars indicate the target genes of miR-148a-3p and miR-128-3p altered by acute subanesthetic ketamine treatment. Genes predicted to be targeted by miR-148a-3p are marked in red, and genes predicted to be targeted by miR-128-3p are marked in blue. Genes predicted to be targeted by both miRNAs are marked in purple.

Among the five ketamine-responsive miRNAs, miR-148a-3p and miR-128-3p target the largest number of genes involved in ubiquitin-mediated proteolysis (Fig 3A). Thus, miR-148a-3p and miR-128-3p were selected for further validation through miRNA qPCR. miR-148a-3p showed increased expression while miR-128-3p showed decreased expression, consistent with the miRNA microarray analysis (Fig 3B and 3C). Fig 4 shows the KEGG-generated ubiquitin-mediated proteolysis pathway with genes putatively targeted by miR-148a-3p and miR-128-3p. Specifically, genes involved in the activity of E2 ubiquitin-conjugating enzyme (E2) and multi subunit RING-finger type E3 ubiquitin ligase (E3) were predicted to be the main targets of miR-148a-3p and miR-128-3p, which suggests that acute subanesthetic ketamine treatment plays a unique and specified role in prefrontal ubiquitin proteasome system.

## Discussion

**Fig 5:**
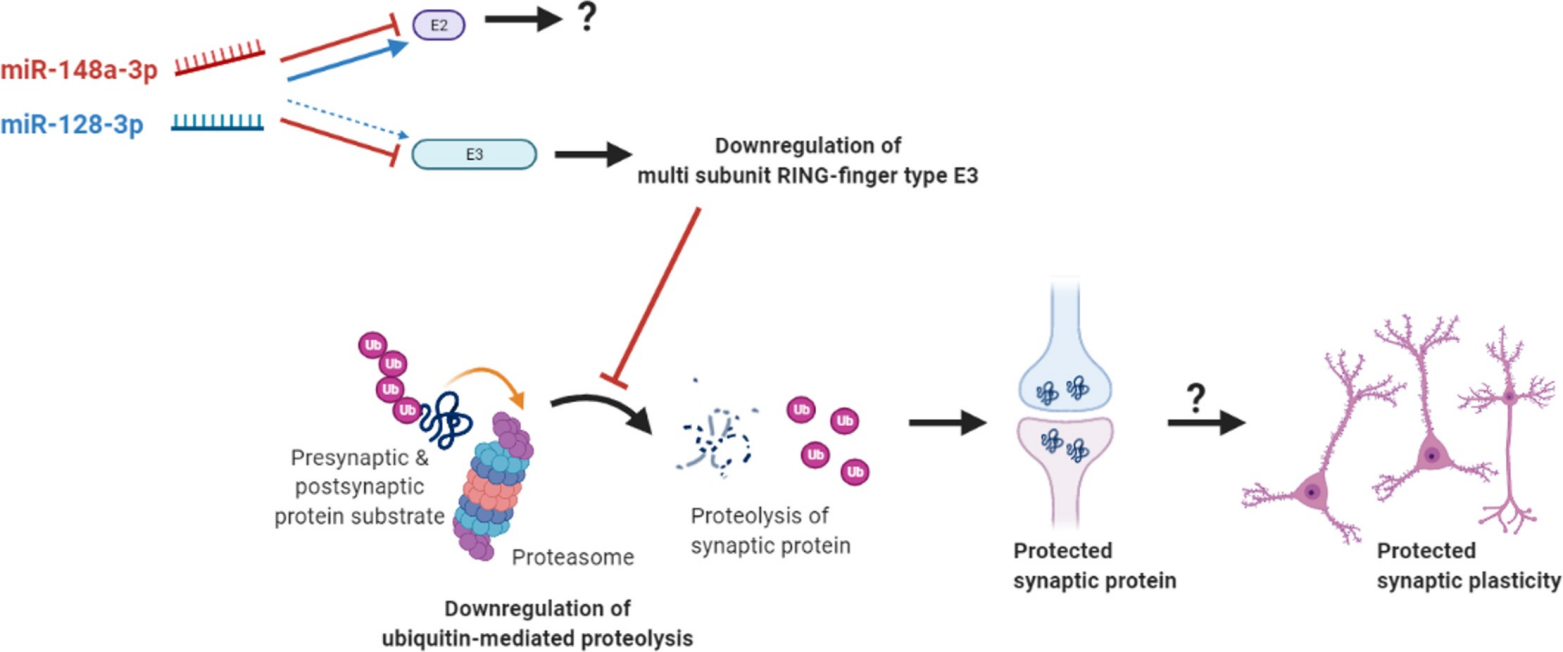
A hypothetical schematic model of the mode of action for acute subanesthetic ketamine treatment mediated by prefrontal miRNAs. Acute subanesthetic ketamine rapidly modified the expression patterns of miR-148a-3p and miR-128-3p in the mPFC, which may lead to the alteration of the genes related to E2 and multi subunit RING-finger type E3. The ketamine-responsive miRNAs are expected to influence ubiquitin-mediated proteolysis of synaptic proteins, which would affect prefrontal synaptic plasticity and exert acute subanesthetic ketamine effects.

In this study, we focused on the mPFC miRNAs altered by acute subanesthetic ketamine treatment in mice. A total of five miRNAs exhibited differential expression patterns in the mPFC of ketamine-treated mice. These five miRNAs were predicted to target a total of 781 genes, and bioinformatics analyses simultaneously predicted that these ketamine-associated genes are involved in ubiquitin-mediated proteolysis. These results indicate that the prefrontal miRNA-dependent regulation of ubiquitin-mediated proteolysis is a mode of action by which acute administration of subanesthetic ketamine acts. Validation experiments revealed that miR-148a-3p and miR-128-3p are rapidly and differentially regulated in the mPFC of ketamine-administered mice, potentially leading to the alteration of the genes related to E2 and multi subunit RING-finger type E3 and thereby regulating ubiquitin-mediated proteolysis of presynaptic as well as postsynaptic proteins that affect prefrontal synaptic plasticity to exert the effects mediated by acute subanesthetic ketamine (Fig 5). Interestingly, as E2-related genes are concurrently targeted by miR-148a-3p and miR-128-3p, it is elusive how the two miRNAs will eventually affect the expression of E2. On the other hand, multi subunit RING-finger type E3-related genes are dominantly targeted by miR-148a-3p, which is predicted to inhibit ubiquitin-mediated proteolysis.

Although miR-148a-3p is known for its role in gastric cancer [37, 38], there is limited information on its role in the brain. Muiños-Gimeno et al. showed that miR-148a is associated with panic disorder phenotypes and represses a candidate gene of panic disorder [39], implying that miR-148a-3p can be a critical regulator of brain diseases. Furthermore, a negative correlation between ubiquitin-mediated proteolysis inducer HOTAIR and miR-148a was found in cervical cancer tissues [40] and the indirect regulation of E3 via miR-148a was reported in prostate cancer cells [41], which indicates that the genes involved in ubiquitin-mediated proteolysis can be targeted by miR-148a-3p. Importantly, our data predicted that miR-148a-3p targets Skp1 and Cul5, which are multi subunit RING-finger type E3 that can regulate synaptic protein. Multi subunit type RING-finger-type E3 SKP1-culling-F-Box protein (SCF) is involved in the ubiquitination and degradation of spine-associated Rap GTPase activating protein (SPAR), a postsynaptic density protein that regulates spine morphogenesis [42].

miR-128-3p is a brain-enriched miRNA found to be elevated in the amygdala of the rat model of learned helplessness [43] as well as in the serum of multiple sclerosis patients [44]. These studies imply that the upregulation of miR-128-3p can negatively affect the brain, resulting in the aggravation of brain diseases. Furthermore, miR-128-3p inhibits the expression of Pellino E3 Ubiquitin Protein Ligase Family Member 3 (PELI3) [45] and SMAD-specific E3 ubiquitin protein ligase 2 (SMURF2) [46], which suggests that miR-128-3p regulates ubiquitin-mediated proteolysis pathway.

Ubiquitin is a regulatory protein that plays an important role in endogenous protein degradation by mediating proteolysis. Ubiquitin conjugates with E1 ubiquitin-activating enzyme, and it is then transferred to the E2. The E3 transfers ubiquitin from the E2 to the target protein, which results in degradation of the target protein by the proteasome [47, 48]. The ubiquitin proteasome system plays a number of important roles in the nervous system, including the regulation of synaptic plasticity and protein turnover [49–51]. Interestingly, degradation of synaptic proteins by enhanced ubiquitin-mediated proteolysis can be observed in stress-induced animal models of depression. A study showed that a rat model of chronic stress exhibits E3-mediated enhancement in ubiquitin-mediated proteolysis of AMPAR and NMDAR subunits [52]. The degradation of AMPAR and NMDAR subunits subsequently caused impaired glutamatergic transmission in the prefrontal pyramidal neurons. Also, chronic stress causes tethering of synaptic density-protecting protein Nrf2 with E2, which leads to proteasome degradation of Nrf2 [53].

In line with these findings, we here reported that ketamine-responsive miRNAs and its putative target genes are involved in ubiquitin-mediated proteolysis, more specifically in the activity of E2 and multi subunit RING-finger type E3 (Fig 4). We particularly emphasize the role of multi subunit RING-finger type E3 as it was predicted to be regulated exclusively by miR-148a-3p, the miRNA upregulated by acute subanesthetic ketamine treatment (Fig 3). Interestingly, the decrease in ubiquitin-mediated proteolysis resulting from miR-148-3p-mediated downregulation of multi subunit RING-finger type E3 could protect the ubiquitin substrates that play an important role in long-term potentiation (LTP) induction, which would eventually preserve synaptic plasticity. AMPAR is a ubiquitin substrate critical for LTP. Transgenic mice overexpressing ubiquitin display increased AMPAR ubiquitination and decreased expression of AMPAR subunits [54], which consequently results in decreased excitability and LTP. Thus, we suggest that ketamine-induced decrease in ubiquitin-mediated proteolysis could block the degradation of AMPAR and synaptic density proteins, and eventually exerts a positive effect on synaptic plasticity.

Previous studies have revealed that protein turnover is a critical process for synaptic plasticity [55, 56]. Protein degradation is an essential component for maintaining protein turnover, and ubiquitin-mediated proteolysis plays a crucial role in mediating protein degradation [50]. As a result, a decrease in ubiquitin-mediated proteolysis might inactivate protein turnover and eventually cause deficits in synaptic plasticity. However, other proteolysis mechanisms exist besides ubiquitin-mediated proteolysis, i.e. the ubiquitin-independent proteasomal degradation. Rpn4 is a proteasome transcriptional activator which is degraded by both ubiquitin-dependent and ubiquitin-independent mechanisms [57, 58]. Also, thymidylate synthase can only be degraded by 26S proteasome in a ubiquitin-independent manner [59, 60]. These studies indicate that alternative proteolytic mechanisms may compensate for the ketamine-dependent decrease in ubiquitin-mediated proteolysis, potentially resulting in sufficient protein turnover for synaptic plasticity.

Interestingly, some studies have reported that the antidepressant effect of ketamine is mediated by activation of opioid receptors [61, 62], which subsequently enhances opioid-induced ERK1/2 phosphorylation [63]. Furthermore, it has been shown that opioid receptors are degraded by HECT ubiquitin E3 ligase Smurf2 [64], which suggests that ketamine may exert its effect through opioid receptors by two different mechanisms; by directly activating the opioid receptors or by attenuating the degradation of opioid receptors through miRNA mediated ubiquitin-proteasome system deactivation.

Although our study has focused on the molecular signature of ketamine in the mPFC, ketamine also influences other brain regions including the hippocampus, nucleus accumbens, and amygdala [65–67]. E3 ubiquitin ligase, which our data predicted to be downregulated by ketamine, plays a crucial role for synaptic plasticity in these particular regions. For instance, E3 murine double minute 2 (MDM2) regulates PSD-95 stability in the hippocampus [68], while another E3 anaphase promoting complex/cyclosome (APC/C) has been implicated in long-lasting synaptic plasticity in the amygdala [69]. Additionally, studies have revealed that E2 Bendless and UbcD1 regulate synapse formation and dendritic pruning in the Drosophila [70, 71]. Collectively, these data indicate that ketamine may also be responsible for the miRNA-mediated downregulation of ubiquitin-mediated proteolysis in other brain regions, which should be addressed in future studies. Also, the relationship between E2 and synaptic plasticity in the ketamine-responsive brain regions may be interesting to explore in the future.

Our study demonstrated that acute subanesthetic ketamine administration is predicted to inhibit prefrontal ubiquitin-mediated proteolysis pathway through miRNAs, which could underlie the fast-acting antidepressant effects of ketamine. This notion is further supported by a recent finding that ketamine exerts its antidepressant effects via weakening of ubiquitin ligase activity and subsequently enhanced mTOR signaling [72]. However, ketamine was also found to increase the ubiquitination of Notch to disrupt downstream targets of the Notch signaling pathway in the Xenopus model of neurodevelopment [73], surfacing a potential controversy in the ubiquitination-mediated mechanism of ketamine. Thus, these findings demand additional research to clarify the biological impact of ketamine on ubiquitin-mediated proteolysis *in vivo*. Moreover, the ketamine-responsive miRNAs, miR-148a-3p and miR-128-3p, as well as the ubiquitin-mediated proteolysis pathway should be explored further in the context of the depression disorder.

## Conclusions

In this study, we aimed to reveal the mode of action associated with acute subanesthetic ketamine treatment through miRNA profiling and bioinformatics analyses in the mPFC of mice. Interestingly, the genes putatively targeted by ketamine-responsive miRNAs were primarily involved in ubiquitin-mediated proteolysis, which we propose as a novel mode of action by the acute subanesthetic ketamine. Validation experiments revealed that the differential expression patterns of miR-148a-3p and miR-128-3p are consistent with miRNA microarray data, and that the two miRNAs preferentially target the ubiquitin ligases E2 and multi subunit RING-finger type E3. Particularly, multi subunit RING-finger type E3 was predicted to be exclusively targeted by miR-148a-3p, which may result in downregulation of the genes associated with ubiquitin-mediated proteolysis. Thus, our data indicate that acute subanesthetic ketamine may exert its effects through the miRNA-dependent regulation of ubiquitin-mediated proteolysis.

## Supporting information

S1 Table A total of 781 genes were found to be putatively targeted by the ketamine-responsive miRNAs. (DOCX)
