# Supplementary material for: Subanesthetic ketamine rapidly alters medial prefrontal miRNAs involved in ubiquitin-mediated proteolysis
Source: PLoS One. 2021 Aug 26;16(8):e0256390. doi: 10.1371/journal.pone.0256390 (PMC8389495; doi:10.1371/journal.pone.0256390)
Supplement: S1 Table — (DOCX) [file pone.0256390.s001.docx]

**S1 Table. A total of 781 genes were found to be putatively targeted by the ketamine-responsive miRNAs.**

|  | Gene Name | | | | | | | | | | | | | | |
| --- | --- | --- | --- | --- | --- | --- | --- | --- | --- | --- | --- | --- | --- | --- | --- |
| Upregulated genes | Szrd1 | Adora2b | Anks1 | Nova1 | Notch2 | Hipk2 | Prkd1 | Phb | Ruvbl2 | Clpp | Pdia5 | Ube2e2 | Matn3 | Poglut1 | Ank2 |
|  | Plk2 | Olfr13 | Dcun1d4 | Rab20 | Rps6ka5 | Sorl1 | Vegfc | Hoxa13 | Ypel3 | H3f3b | Zbtb20 | Prkx | Ttc39a | Onecut2 | Ak2 |
|  | Plagl1 | Ppp1cc | Trim23 | Rundc3a | Ube2n | Tmsb10 | Mipol1 | Cacng2 | Pogz | Fam184a | Gltp | Zfp462 | Lsm1 | Nrep | Sh3bgrl2 |
|  | Plscr1 | Lin28a | Tmx1 | Ywhab | Grin2d | Tspo2 | Phf6 | Slc6a1 | Csdc2 | Mmd | Casc3 | Orc5 | Trim12c | Sgpp1 | Kcnk2 |
|  | Mdfi | Sp1 | Paip2 | Fam134c | Slc5a3 | Samd10 | Rap1b | Ppp4c | Gatad2a | Gpx8 | Lbh | Camk1d | Agbl3 | Nxt2 | Megf11 |
|  | Ubr5 | Snap25 | Car10 | Unc13c | Mapk14 | Ugt8a | Arrdc4 | Gtf2a2 | Tmem240 | Acvr1 | Mob1b | A930004D18Rik | BC030336 | Mospd3 | Cabp1 |
|  | Atl3 | Snx12 | Ube2w | Ngp | Sgms1 | Sec61a1 | Vangl1 | Eml1 | Usp42 | Nrbf2 | Bend4 | Aqp11 | Galnt3 | Arhgap12 | Rnf38 |
|  | Jag1 | Thrb | Ostf1 | Csf1 | Rgl2 | Tmem64 | Ppif | Rnf139 | Kcnk10 | Mroh3 | Pard6b | Bmi1 | Ngfr | Tnpo1 | Isl1 |
|  | Rasgef1b | Sall4 | Sh2d3c | Reln | Fam78a | Ccdc71 | Sssca1 | Hlx | Fbxo33 | Nfx1 | Diras2 | Slc35f3 | Tceanc2 | Stim2 | Rab34 |
|  | E130309F12Rik | Rgs8 | Iglon5 | Diras1 | Tgif1 | Glipr2 | Acad12 | Slc24a4 | Hoxa5 | Cyp39a1 | Plagl2 | Apba2 | Arhgap21 | Smad9 | Sec24a |
|  | Rnf125 | Nipal4 | Gabra6 | Mturn | En1 | Abhd17b | Zfp385a | Stx16 | Btg2 | Slco5a1 | Sp2 | Rffl | Rnf144a | Bgn | Neurod6 |
|  | Slc35f1 | Gm16039 | Car7 | Dll4 | Pparg | Naa50 | Igj | Chrm2 | Ing5 | Nhs | Trim5 | Lpar6 | Kbtbd8 | Ccdc50 | Arl8b |
|  | Zfp36l1 | Ccdc92 | Naa15 | Fbxw7 | Stk32a | Samd12 | Fam155a | Stk32b | Socs6 | Tmeff1 | Galnt7 | Fam126b | Agfg1 | Fam84b | Usp49 |
|  | Dtx4 | Aff4 | Vav3 | Kcnd3 | Mpped1 | Dcaf7 | Erlec1 | Ikbke | Phlpp2 | 1700008O03Rik | C130026L21Rik | Arhgef11 | Pde7b | Hao1 |  |
| Downregulated genes | Meox2 | Arl6ip1 | Snn | Gadd45a | Eogt | Tmem54 | Arrdc3 | Nptn | S1pr1 | Szrd1 | Npepl1 | Cdk19 | D1Ertd622e | Ucp3 | B4galt5 |
|  | Cckbr | Gfra4 | Rab34 | Qk | Maf1 | Sgcb | Stk38l | Cabp7 | Ubap2l | Fez2 | Stam | Arfip1 | Uhmk1 | Mmd | Osbpl11 |
|  | Vamp5 | Sgms1 | Dock6 | Gm9923 | Prune | Errfi1 | Arap2 | Nat14 | Agfg1 | Zfp110 | Plaa | Slc25a53 | Chchd4 | Barhl2 | Lgalsl |
|  | Mtmr12 | Tgfa | Bicc1 | Tnrc6a | Rock1 | C1galt1 | Tpp1 | Elavl4 | Pnpla6 | Bcl2l11 | Ppp6r1 | Cdk5r1 | Itga5 | Rab14 | Mras |
|  | Sos2 | 8030462N17Rik | Mitf | Usp32 | Mtmr14 | Mnt | Rictor | Ywhab | Tnrc6c | Pea15a | Csf1 | C330018D20Rik | Dpp4 | Cyth3 | Pdia3 |
|  | B630005N14Rik | Phactr2 | Ythdc2 | Btbd3 | Tmsb4x | Ajuba | Lbr | Ubxn1 | Wasl | Nptx1 | Gpatch8 | Bbc3 | Kat7 | Gm5065 | H2afy |
|  | Trak2 | Cul5 | Stx3 | Inhbb | Dgcr8 | St18 | Jph3 | Snx27 | Dnmt1 | Nras | Ube2d3 | Cand1 | Fxr1 | Mdfic | Fam104a |
|  | Fam43a | Abcb7 | Hecw2 | Mllt10 | Adam10 | Abca1 | Esr1 | Hmg20a | Dcp2 | B230219D22Rik | Med12l | Rph3al | Itga9 | Pi4k2a | Gap43 |
|  | Ube2d1 | Slc24a3 | Nova1 | D5Ertd579e | Ino80 | Robo1 | Epn2 | AI118078 | BC005537 | Fbxl19 | Tbp | Gpm6a | Nog | Ube2w | Klf6 |
|  | Sms | Naa15 | Eps15 | Mmp15 | Bmp2k | Srsf11 | Cds1 | Fmr1 | Mgat5 | Ism1 | Fam122a | Kmt2a | Neurl4 | Cntn4 | Esco1 |
|  | Prkaa1 | Itsn2 | Rnf38 | Gm10300 | Mafb | St8sia3 | Crtc3 | Esrrg | Gabarapl2 | Slc25a44 | Kcns2 | Skp1a | Txnip | Rab12 | Ypel3 |
|  | Maf | Lrrc41 | Taf4b | Ormdl1 | Ccnf | Itga11 | Ulk3 | Nrp1 | Cnot6 | Ahdc1 | Ptpra | Ago4 | Hsp90b1 | Dynll2 | Wnt10b |
|  | Cux1 | Serpine1 | Ppp1r9a | Usp48 | Znrf1 | March2 | Emx2 | Mpped2 | Arl8b | Zfp804a | Kcna1 | 5430435G22Rik | Dcun1d3 | Ppp1r10 | Cyb5r4 |
|  | Prr5l | Arhgap21 | Foxf1 | Nol4 | E2f7 | Tmed7 | Fbn1 | Btaf1 | Tgif2 | Col2a1 | Xpo4 | D230025D16Rik | Clock | Zadh2 | Fam184a |
|  | Skida1 | Otud4 | Ddx6 | Klf4 | Ndp | Stradb | Mtmr10 | Ssr1 | Ppp1cb | Akap1 | Chuk | Stox2 | Stxbp5 | Cdk8 | Cdc25b |
|  | Acvr1 | Mtf1 | Mtss1l | Hivep1 | Styx | Elf5 | Rsbn1l | Gpi1 | Rgma | Pdk4 | Rab11b | Usp47 | Prickle2 | Nfya | Lrp4 |
|  | Dmxl1 | Sos1 | Zfp97 | Zfp960 | Gm14420 | Gm6710 | 9830147E19Rik | Gm14431 | Gm14295 | 2410141K09Rik | Zfp850 | Gm14440 | Gm3055 | Zfp781 | Gm14391 |
|  | Gm14326 | Gm14322 | Gm14306 | Gm5595 | Gm14288 | Zfp951 | Zfp442 | Zfp930 | C030039L03Rik | Gm2381 | Gm14412 | 2210418O10Rik | Gm14296 | B230307C23Rik | 5730507C01Rik |
|  | Zfp780b | Zfp937 | Zfp120 | Gm17067 | Zfp607 | Zfp619 | 6720489N17Rik | Gm7221 | Zfp869 | RP23-293C19.2 | Osbpl3 | RP23-133G16.2 | Zfp872 | Zfp14 | Gskip |
|  | Zfp825 | Rnf34 | RP24-392D11.2 | 1700066M21Rik | Rorb | Esm1 | Rbbp7 | Med8 | Phip | Gabra1 | Il1a | Srsf7 | Hmbs | Lyrm1 | Crebrf |
|  | Ccl7 | 4921524J17Rik | Atp1b1 | Yod1 | Rbm3 | Ddx55 | Zfp36l1 | Afg3l2 | Cpne2 | E2f5 | Tcerg1 | Rassf1 | Zfand5 | Txndc12 | Zfp867 |
|  | Tmem165 | Entpd6 | Syne1 | Dcun1d1 | Ppip5k2 | Gm5113 | Ap1s3 | Mturn | Slitrk1 | Klf15 | Slc9a3 | Chic1 | Plekhj1 | Synpr | Trim2 |
|  | Evx1 | Phtf2 | Trak1 | Rlf | Npepps | Hoxa11 | Cpeb4 | Gata6 | Hmgb2 | Gpbp1 | Tnfrsf11b | Gm14325 | Ippk | Sall4 | Larp4 |
|  | Hoxa1 | Cks1b | Dlgap2 | Prkcd | Ss18l1 | Il7 | Epc2 | Pnrc2 | Gm4791 | Tnf | Gm6871 | Dnaja4 | Wsb1 | Bhlhe40 | Hmgb1 |
|  | Nek7 | Ptbp2 | Msantd3 | Lin28b | Zfand1 | Pi4k2b | Zfp286 | Tmem132d | Slc25a37 | Zfand6 | Pgap1 | Prtg | Cbx4 | Anapc16 | Mlf1 |
|  | Bclaf1 | Brd1 | Rnmt | Rbm47 | Nab1 | Pam | Fign | Tbpl1 | Hlf | Hey2 | Evi2a | Itgb8 | Atp2b1 | Cul3 | Ubl3 |
|  | Timp3 | Cnksr3 | Ddx3x | Lmo1 | Zfp820 | Sowaha | Aak1 | Slmap | C77370 | Taf13 | Gm14327 | Derl1 | Mboat2 | RP23-408P8.1 | 1810011O10Rik |
|  | Gfpt1 | Ccnk | Tom1l1 | Lhx9 | Elmod2 | Gxylt1 | Ddah1 | Ubash3b | Dazap1 | Foxj3 | Eya2 | Syt5 | Otx2 | Klb | Snrk |
|  | Nr2c2 | Elovl7 | Tmem98 | Isl1 | Cnrip1 | Cxxc5 | Slc31a1 | Sox14 | Rbms3 | Bbs4 | Sdk1 | Rassf3 | Cc2d1a | Nipa2 | Erg |
|  | Sp4 | Crlf3 | Ror1 | Fzd4 | T | Cxxc4 | P4ha1 | Mms19 | Reck | Inpp5j | Atf7ip2 | Rbm24 | Laptm4a | Gpr26 | Gltscr1l |
|  | Camk1d | Mstn | Sav1 | Slk | Cacul1 | Slc41a1 | Dnajc6 | Thrb | Fbxo3 | Ube2n | Pigg | Rab35 | Kcnj2 | Pigr | Col9a1 |
|  | Fgf9 | Mecom | Kcnh8 | Sh3d19 | Atg14 | Sox5 | Ube2z | Klf16 | Tsc22d2 | Has3 | Sox6 | Ubr1 | Efnb2 | Dph6 | Trnp1 |
|  | Map9 | Ccdc177 | Kbtbd8 | Klf3 | Abhd13 | Esr2 | Elk1 | Nipal2 | Upf3b | Pkig | CDR1as | Pole4 | Sp1 | Mrgprf | Ksr1 |
|  | Snca | Gm7120 | Trmt13 | Gtf2a1 | Spata2 | Nrep | Bloc1s4 | 0610007P14Rik | Ghitm | Ost4 | 4933426M11Rik | H2-Ob | Plp2 | Champ1 | Raf1 |
|  | Trpm7 | Cct4 | Pan2 | Wipf2 | Cnppd1 | Fam168b | 3110001I22Rik | Rgs8 | Strn3 | Nr1h2 | Gjc1 | Tmed9 | Ckap4 | Slc5a3 | Fndc4 |
|  | 1110008F13Rik | Chsy3 | Mknk1 | Dcstamp | Parp1 | Pfn2 | Pax6 | Gal3st3 | Rnf141 | Kif13a | Atf7 | Gltpd1 | Arid4a | Ublcp1 | Igsf8 |
|  | Fam53c | Ankrd12 | Smim12 | Cacng7 | Gmeb1 | Herpud2 | Cxcl2 | Crk | Ccdc120 | March11 | Fgf1 | Cnn3 | Rsbn1 | Vps26a | Ptgfrn |
|  | Gm10118 | Wdr47 | Smyd5 | Galnt3 | Vimp | Ppif | Pbx3 | Iglon5 | Ide | Lrtm2 | Marf1 | Ezh1 | Rbms1 | Arrb1 | Plcb1 |
|  | Cnot8 | Rb1 | Pde4d | Naa60 | Irs2 | Mafg | Serp1 | Calr | Flrt2 | Bcorl1 | Zfp768 | Ccnt2 | Hells | Slc25a15 | Tmub2 |
|  | Rgs7bp | Ablim1 | Megf9 | Smndc1 | Eif3j1 | Zbtb22 | Atxn7 | Hs3st5 | Lemd3 | Capza1 | Naif1 | Ccdc43 | Zc3h4 | Tfrc | Fam83a |
|  | Rnf144a | Pacsin1 | Hcn1 | Vma21 |  |  |  |  |  |  |  |  |  |  |  |

209 genes are upregulated and 604 genes are downregulated by ketamine-responsive miRNAs. 32 genes were commonly detected in both upregulated and downregulated genes.
